# Association of CCR6 functional polymorphisms with Primary Biliary Cholangitis

**DOI:** 10.1016/j.jtauto.2024.100234

**Published:** 2024-02-15

**Authors:** Mingming Zhang, Zhuye Qin, Yexi Huang, Wenyan Tian, You Li, Chan Wang, Weifeng Zhao, Yaping Dai, Xingjuan Shi, M. Eric Gershwin, Xiong Ma, Meilin Wang, Xiangdong Liu, Weichang Chen, Fang Qiu

**Affiliations:** a Key Laboratory of Developmental Genes and Human Diseases, School of Life Science and Technology, Southeast University, Nanjing, Jiangsu, 210096, China; b Department of Laboratory Medicine, Southeast University Hospital, Southeast University, Nanjing, Jiangsu, 210096, China; c Department of Gastroenterology, The First Affiliated Hospital of Soochow University, Suzhou, Jiangsu, 215006, China; d Department of Gastroenterology and Hepatology, Shanghai Institute of Digestive Diseases, Shanghai Jiao Tong University School of Medicine Affiliated Renji Hospital, Shanghai, 200001, China; e Institute of Translational Medicine, Yangzhou University Medical College, Yangzhou, Jiangsu, 225009, China; f Department of Hepatology, The First Affiliated Hospital of Soochow University, Suzhou, Jiangsu, 215006, China; g Department of Laboratory Medicine, The Fifth People's Hospital of Wuxi, Wuxi, Jiangsu, 214000, China; h Division of Rheumatology, Allergy and Clinical Immunology, University of California at Davis School of Medicine, Davis, CA, 95616, USA; i Department of Genetic Toxicology, The Key Laboratory of Modern Toxicology of Ministry of Education, Center for Global Health, Nanjing Medical University School of Public Health, Nanjing, Jiangsu, 210029, China; j Department of Laboratory Medicine, The Fourth Affiliated Hospital of Nanjing Medical University, Nanjing, Jiangsu, 210031, China

**Keywords:** Primary biliary cholangitis, Han Chinese, CC chemokine receptor 6, CC chemokine ligand 20, Single nucleotide polymorphism

## Abstract

The biliary epithelial cells release CC chemokine receptor 6 (*CCR6*) ligand 20 (*CCL20*), leading to recruitment of CCR6^+^ T cells and subsequent infiltration into the biliary epithelium in primary biliary cholangitis patients. Previous genome-wide multi-national meta-analysis, including our Han Chinese cohort, showed significant association of *CCR6* and *CCL20* single nucleotide polymorphisms (SNP) with PBC. We report here that significantly associated SNPs, identified in the *CCR6* locus based on our Han Chinese genome-wide association study, can be separated into “protective” and “risk” groups, but only “risk” SNPs were confirmed using a separate Han Chinese PBC cohort. Only weak association of *CCL20* SNPs was observed in Han Chinese PBC cohorts. Fine-mapping and logistical analysis identified a previously defined functional variant that, leads to increased *CCR6* expression, which contributed to increased genetic susceptibility to PBC in Han Chinese cohort.

## Highlights

• *CCR6* locus is significantly associated with PBC susceptibility in Han Chinese cohorts.
• Expression of CCR6 was significantly elevated in the livers of PBC patients.
• *CCL*20 locus is weakly associated with the PBC susceptibility in Han Chinese cohorts.
• Functional variants associated with increased *CCR6* expression may lead to increased intrahepatic bile duct inflammation.

## 1 Introduction

Primary biliary cholangitis (PBC) is a chronic progressive cholestatic autoimmune liver disease that tendentiously injures small intrahepatic bile ducts, and may lead to cirrhosis without effective treatment [1]. The pathogenesis of PBC is complicated and multifactorial. A large number of studies confirmed the strong genetic predisposition to PBC [2]. Previous genome-wide association studies (GWAS) in European, Japanese, and Han Chinese population identified numbers of PBC susceptibility loci, including human leukocyte antigen (HLA) and non-HLA loci [[3], [4], [5], [6], [7]]. The Han Chinese GWAS analysis of 1126 PBC cases and 4036 healthy controls conducted by our group identified multiple SNPs in C–C chemokine receptor type 6 (*CCR6*) locus with *P* value between 1 × 10^−4^ and 2.04 × 10^−7^, indicating *CCR6* as a potential risk locus for PBC. A subsequent meta-analysis combining European and Japanese data and our data showed several SNPs in *CCR6* locus reaching genome-wide significance [8].

*CCR6* gene is a transmembrane G-protein coupled receptor and expressed mainly on T cells, B cells and immature DC cells [9,10]. *CCR6* serves as the receptor for *CCL20*, a C–C type chemokine. The genome-wide meta-analysis using multiple European cohorts identified a SNP (rs4973341) in *CCL20* locus associated with PBC [11], but rs4973341 and SNPs in its shared haplotype, were not included in the Human OmniZhongHua-8 Beadchip (v1.1), used in the Han Chinese GWAS study.

In this report, we carried out a confirmational analysis of *CCR6* and *CCL20* loci with newly collected PBC cohorts and performed linkage disequilibrium and conditional regression analyses to pinpoint potential SNPs in *CCR6* locus directly associated with PBC. We also performed genotyping analysis of rs10933215, which is in complete linkage disequilibrium with rs4973341 in Chinese cohorts. and we further analyzed the interaction among significant SNPs observed in *CCR*6 and *CCL*20.

## 2 Materials and methods

### 2.1 Study subjects

The study used a two-stage approach involving GWAS-based discovery and replication using TaqMan SNP genotyping in an independent population. All patients with PBC were diagnosed in accordance with the criteria recommended by the American Association for the Study of Liver Diseases [12]. All PBC serum samples were tested for AMA-M2, anti-gp210 and anti-sp100 antibodies using in-house developed ELISA tests. The detailed diagnosis and patient inclusion have been described in our previous publication [13]. Among them, 1126 PBC patients and 4036 healthy controls were reported in our previous GWAS [7,14,15]. A total of 2093 PBC patients, 1129 healthy controls were from the Jiangsu Provincial PBC Collaborative Groups, and 2207 extra controls from the Colorectal Cancer Research Project were included in the replication cohort [16].

### 2.2 SNP genotyping

Genomic DNA is extracted from blood samples. SNP genotyping in the replication study was performed by TaqMan assay for rs12529876, rs4710181 and rs6905911. The primer and probe sequences are listed in Table S1. The TaqMan assay was performed using a Bio-Rad CFX Manager Real-Time PCR system in 10 μL of reaction mixtures containing 1 × PCR buffer, 2 mM deoxyribonucleotide triphosphate mix, 2 μM of each primer, 3 μM of labeled probe, 0.25 U Taq HS polymerase (Takara), 5 ng of genomic DNA, and 6.15 μL deionized water. PCR conditions were as follows: 95 °C for 10 min, followed by 40 cycles of 95 °C for 30 s and 60 °C or 62 °C for 30 s. To sequence for CCR6DNP polymorphism, PCR reaction samples were sent to the company for Sanger sequencing. Primer sequences for sequencing are as follows: 5′-CGCTTTTGACTAGCACTCGA-3′, 5′-TTGTTCATCCCAACCTCCCT-3’. Product length: 264bp.

### 2.3 Immunohistochemistry

Paraffin-embedded liver tissues, derived from ultrasound-guided needle liver biopsies of 30 PBC patients, 30 autoimmune liver hepatitis (AIH) patients, 14 non-alcoholic fatty liver disease (NAFLD) patients, 14 chronic hepatitis B (CHB) patients, and 6 healthy controls (HC), were studied. Sections were stained with either hematoxylin and eosin (H&E) or Masson and were independently reviewed “blindly”. Inflammatory degrees and fibrotic stages were graded according to the Scheuer scoring system. The six healthy liver tissues were collected from donors whose livers were subsequently used for liver transplantation. Immunohistochemistry imaging of CCR6 staining of human liver tissues was performed on a Leica Bond system (Leica, Germany) using the standard protocol. The liver sections were pre-treated using heat-mediated antigen retrieval with sodium citrate buffer (pH 6, epitope retrieval solution 1) for 20 min followed by incubation with an anti-CCR6 antibody (1:200 dilution, ab93086, Abcam) for 20 min at room temperature and then detected using a horse radish peroxidase-conjugated compact polymer-system. 3,3′-diaminobenzidinewas used as the chromogen. The liver sections were then counterstained with hematoxylin. All the sections were visualized using light microscopy (Olympus, Japan), and five fields were randomly selected for each section. The numbers of CCR6-positive cells were quantified at 40 × 10 magnification. All the samples were examined by a hepatic pathologist, and all data are reported as mean ± standard error (s.e.).

### 2.4 Statistical analysis

Binary logistic regression analysis with SPSS 23.0 (SPSS, Inc., Chicago, IL) was used to determine *P* values, odds ratios (OR), and confidence intervals (CI) for the association between SNPs for the validation analysis. Stata12.0 (Stata Corporation, College Station, TX) software was used to conduct a meta-analysis using a random effect model for the two distinct case-control cohorts. Conditional logistical regression analysis was performed by PLINK 1.9 (http://pngu.mgh.harvard.edu/purcell/plink/) [17]. The Mann–Whitney *U* test was used to evaluate differences in continuous variables, a *P* value of <0.05 was considered statistically significant, correlations were determined using the Spearman's correlation coefficient, all analyses were two-tailed and performed using Prism software Version 6.0 (Graphpad Software, La Jolla, CA, USA). Linkage disequilibrium analysis was performed using Haploview 4.2 [18]. Gene and protein expression data in cells and tissues were obtained from the Human Protein Atlas (https://www.proteinatlas.org/). Both SNP information and linkage disequilibrium data were downloaded from the Ensembl Database (https://grch37.ensembl.org/index.html). Functional annotation of SNPs was carried out using HaploReg v4.2 database (https://pubs.broadinstitute.org/mammals/haploreg/haploreg.php) [19] and the Regulome DB v2.2 database (https://regulomedb.org/regulome-search/) [20,21].

## 3 Results

### 3.1 Verification of genetic association of polymorphisms in *CCR6* locus with PBC

**Fig. 1:**
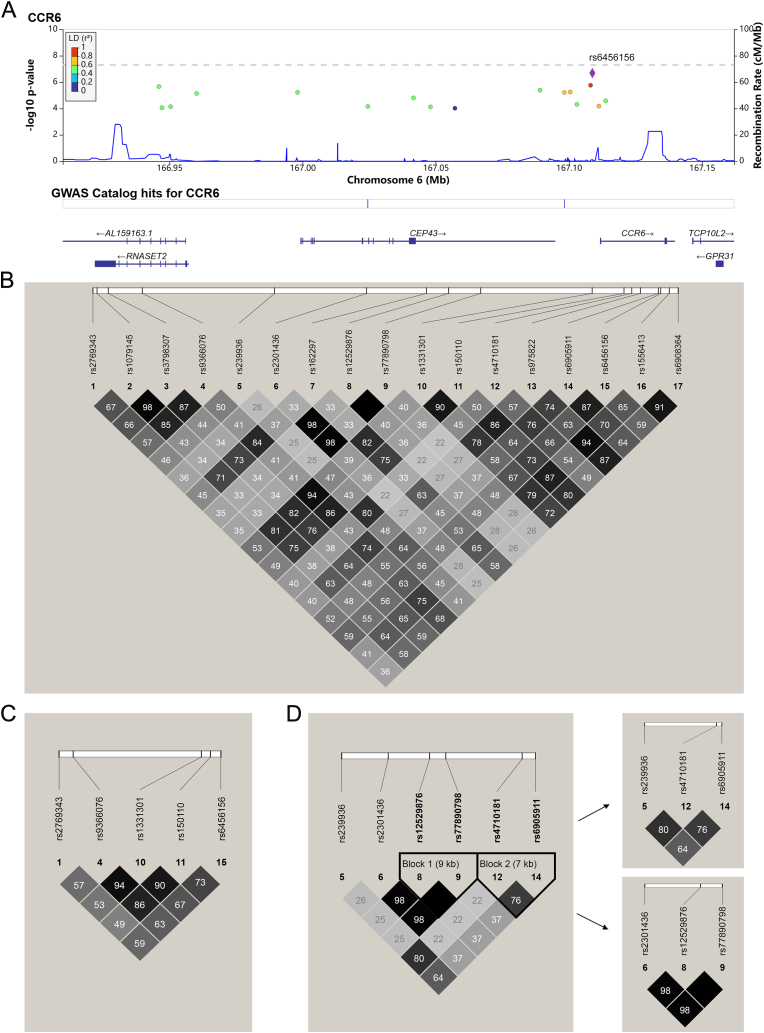
Locus specific plots and LD structure for SNPs in *CCR6* locus based on the GWAS data. (A) The LocusZoom plots of GWAS *P* value and LD (r^2^) of 17 significant SNPs with the most significant SNP are shown by the color codes (see legend) depending on their expected degree of correlation (r^2^) with the top SNP. (B)The LD plot of 17 significant SNPs in *CCR6* locus which *P* value less than 1 × 10^−4^ in Han Chinese. (C) The LD plot of 5 protective SNPs in *CCR6* locus. (D) The LD plot of 6 risk SNPs in *CCR6* locus. Risk SNPs were grouped into two blocks based on linkage disequilibrium. All SNPs were analyzed by Haploview v4.2. Darker color denotes a higher correlation between markers (r^2^). The plot of pair-wise LD analysis and haplotype block of SNPs of *CCR6* was drawn by using 1126 Han Chinese PBC samples from GWAS data. (For interpretation of the references to color in this figure legend, the reader is referred to the Web version of this article.)

In previous study, we performed a genome-wide analysis using the Human OmniZhongHua-8 Beadchip (v1.1) on 1126 PBC cases and 4036 healthy controls [7]. There were seventeen SNPs in the *CCR6* locus (chr:6q27) with *P* values ≤ 1 × 10^−4^ (Fig. 1A and Fig. S1). These seventeen SNPs were divided into two groups, which played a “risk” and “protective” roles, respectively (Table S2). There is no linkage association between these two types of SNPs (Fig. 1B, C, 1D). Among the protective SNPs, the most significant SNP is rs6456156, with a *P* value of 2.04 × 10^−7^. In the replication study, we genotyped rs6456156 in additional 907 PBC cases and 2127 controls, but the *P* value did not reach statistical significance, *P* = 0.128 [7].

Further linkage disequilibrium (LD) analysis among the “risk” SNPs revealed two sets of LD blocks represented by rs6905911 and rs12529876, respectively (Fig. 1D). We performed replication study for rs12529876 and rs4710181 and rs6905911 in a separated 1885 PBC cases and 3127 normal controls. The observed allelic associations were *P* = 2.76 × 10^−5^ (OR = 1.18, 95% CI = 1.10–1.27) for rs12529876, *P* = 6.99 × 10^−4^ (OR = 1.14, 95% CI = 1.06–1.23) for rs4710181, and *P* = 5.54 × 10^−5^ (OR = 1.17, 95% CI = 1.08–1.26) for rs6905911, respectively (Table S3). Subsequent meta-analysis combining GWAS and validation data for rs12529876, rs4710181, and rs6905911 showed *P* value of 5.59 × 10^−9^ (OR = 1.22, 95% CI = 1.14–1.30), 2.02 × 10^−7^ (OR = 1.19, 95% CI = 1.11–1.27), and 2.68 × 10^−9^ (OR = 1.22, 95% CI = 1.14–1.30), respectively, reaching GWAS significant levels (Table 1). These results confirmed that the *CCR*6 locus is indeed associated with PBC susceptibility in Chinese Han cohorts.

**Table 1:** Meta-analysis of SNPs in the *CCR6* locus associated with susceptibility to PBC.

SNP	Minor Allele	GWAS cohort				Replication cohort				Meta-analysis	
		Case (n = 1126)	Control (n = 4036)	*P*-value	OR	Case (n = 1960)	Control (n = 3316)	*P*-value	OR	*P*-value	OR
		MAF	MAF		(95% CI)	MAF	MAF		(95% CI)		(95% CI)
rs12529876	A	0.429	0.382	7.51 × 10^−5^	1.23 (1.12–1.35)	0.416	0.377	2.76 × 10^−5^	1.18 (1.10–1.27)	5.59 × 10^−9^	1.22 (1.14–1.30)
rs4710181	C	0.478	0.424	5.62 × 10^−6^	1.24 (1.13–1.37)	0.452	0.419	6.99 × 10^−4^	1.14 (1.06–1.23)	2.02 × 10^−7^	1.19 (1.11–1.27)
rs6905911	T	0.542	0.485	1.72 × 10^−6^	1.26 (1.15–1.38)	0.518	0.481	5.54 × 10^−5^	1.17 (1.08–1.26)	2.68 × 10^−9^	1.22 (1.14–1.30)

MAF, minor allele frequency; GWAS, genome-wide association study; OR, Odds ratio; 95% CI, 95% Confidence interval.

### 3.2 The functional CCR6DNP variant is associated with PBC susceptibility

To further investigate potential causal variation, we sought to determine whether the identified significant SNPs were in linkage disequilibrium with the recently discovered functional dinucleotide polymorphism CCR6DNP variant [[22], [23], [24]]. The CCR6DNP variant was genotyped successfully in 918 PBC cases and 1297 healthy controls using Sanger sequencing method. With the allele CG as the reference genotype, allele TG (*P* = 3.72 × 10^−4^, OR = 1.26, 95% CI = 1.11–1.42) was significantly associated with the occurrence of PBC, and allele CA (*P* = 0.607, OR = 1.06, 95% CI = 0.84–1.35) was not significantly different between the two groups (Table 2). The CCR6DNP loci included rs968334 and rs117912866. To identify specific PBC susceptibility loci, PBC association analysis was performed for these two SNPs separately. Rs968334 with allele C as the reference genotype and allele T (*P* = 4.04 × 10^−4^, OR = 1.25, 95% CI = 1.10–1.41) was significantly associated with the occurrence of PBC. Rs117912866 with allele G as the reference genotype and allele A (*P* = 0.753, OR = 0.96, 95% CI = 0.77–1.21) was not significantly different between the two groups. It is apparent that the association of CCR6DNP with PBC was mainly due to the significant difference of rs968334 between PBC patients and controls (Table 2). This conclusion was further confirmed by conditional logistical regression analysis among four significant SNPs (Table 3).

**Table 2:** Genotypic and allelic association analysis of CCR6DNP, rs968334 and rs117912866 in the PBC and control cohort.

SNP	Alleles	PBC (MAF)	Controls (MAF)	OR (95% CI)	*P-*value
CCR6DNP	CG	914 (0.498)	1422 (0.548)	1.0 (ref.)	
	TG	790 (0.430)	979 (0.377)	1.26 (1.11–1.42)	3.72 × 10^−4^
	CA	132 (0.072)	193 (0.074)	1.06 (0.84–1.35)	0.607
rs968334	C	1046 (0.570)	1615 (0.623)	1.0 (ref.)	
	T	790 (0.430)	979 (0.377)	1.25 (1.10–1.41)	4.04 × 10^−4^
rs117912866	G	1704 (0.928)	2401 (0.926)	1.0 (ref.)	
	A	132 (0.072)	193 (0.074）	0.96 (0.77–1.21)	0.753

MAF, minor allele frequency; OR, Odds ratio; 95% CI, 95% Confidence interval.

**Table 3:** The conditional logistic analysis of SNPs associated with PBC.

SNP	Chr	Position	Allele^a^	*P*^b^-value	OR (95% CI)	Cond. *P*-value	Cond. *P*-value	Cond. *P*-value	Cond. *P*-value
						rs12529876	rs4710181	rs6905911	rs968334
rs12529876	6	167048013	A/G	2.76 × 10^−5^	1.18 (1.10–1.27)	NA	0.004	0.035	0.415
rs4710181	6	167100510	C/T	6.99 × 10^−4^	1.14 (1.06–1.23)	0.817	NA	0.234	0.774
rs6905911	6	167108136	T/C	5.54 × 10^−5^	1.17 (1.08–1.26)	0.364	0.013	NA	0.8
rs968334	6	167112608	T/C	4.04 × 10^−4^	1.25 (1.10–1.41)	0.13	0.001	0.021	NA

a Minor/major allele.

b Association *P*-value.

### 3.3 Functional annotations of susceptible variants in *CCR6* locus

Based on Chinese Han South (CHS) and Chinese Han Beijing (CHB) 1000 genomes HapMap data, we summarized SNPs which exhibited strong LD (r^2^ > 0.8) with rs12529876, rs4710181, rs6905911 and rs968334, respectively. RegulomeDB v2.2 and HaploReg v4.2 databases were used for functional annotation of these SNPs (Table S4, Table S5, Table S6 and Table S7). According to the functional annotations, selected SNPs in *CCR6* locus might affect the regulation of transcription. All four SNPs show high scores, with rs12529876, rs4710181 and rs6905911 at “1f”, and rs968334 at “1b”, indicating a higher likelihood of functionality (Table 4).

**Table 4:** Summary of functional annotation of *CCR6* SNPs from RegulomeDB and HaploReg.

chr	pos (hg38)	LD	variant	Promoter^a^	Enhancer^b^	DNAse^c^	Proteins	Motifs	Selected	GENCODE	dbSNP	RegulomeDB rank^f^
		(r^2^)		histone marks	histone marks		bound^d^	changed^e^	eQTL hits	genes	func annot	
6	167048013	1	rs12529876	BLD, GI	BLD, THYM	4 tissues		Sox	11 hits	5.6 kb 3′ of FGFR1OP		1f
6	167100510	1	rs4710181		BLD	BLD	5 bound proteins		5 hits	11 kb 5′ of CCR6		1f
6	167108136	1	rs6905911		BLD			Cdx2, Hsf	3 hits	3.7 kb 5′ of CCR6		1f
6	167112608	1	rs968334	BLD, GI		BLD, BLD		NRSF, Zfp740	7 hits	CCR6	intronic	1b

a Histone modifications of H3K4me1 and H3K27ac.

b Histone modification of H3K4me3.

c The levels of DNase I hypersensitivity.

d The binding of transcription factor.

e The alteration in regulatory motif.

f Functional prediction scores of each SNP by the RegulomeDB database.

### 3.4 Elevated expression of CCR6 in PBC liver lesions

**Fig. 2:**
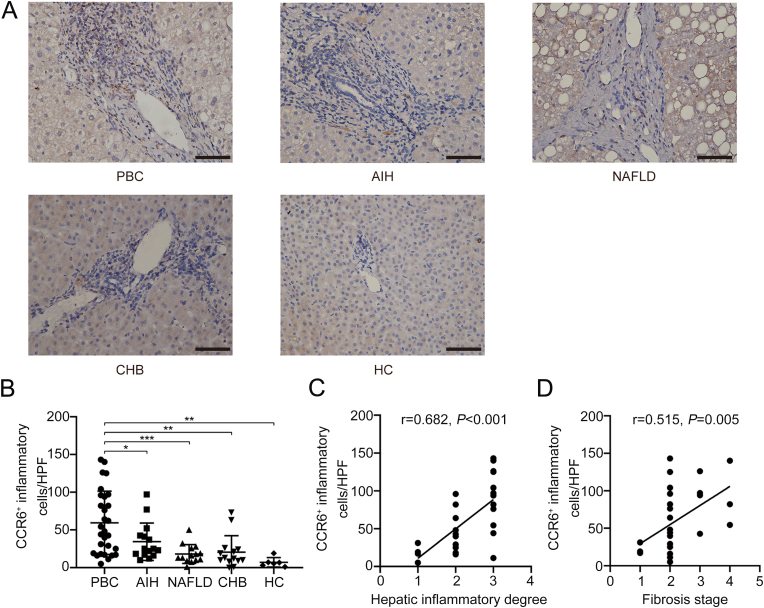
Liver immunohistochemical staining of CCR6. (A) Representative staining images from patients with PBC, AIH, NAFLD, CHB and HC. (B) Quantification of hepatic CCR6^+^ cells in PBC, AIH, NAFLD, CHB and HC, mean ± s. e.m. The frequency of hepatic CCR6^+^ cells is positively correlated with hepatic inflammation degrees (C) and fibrosis stages (D) in PBC (*P < 0.05, **P < 0.01). Scale bars, 20 μm.

To further investigate the potential contribution of deregulated CCR6 to PBC pathogenesis, we conducted immunohistochemical analysis on liver biopsy samples. Compared with AIH patients, NAFLD patients, CHB patients or HC, the expression intensity of CCR6 was significantly elevated in the livers of PBC patients. In particular, CCR6^+^ cells were found to be clustered in inflamed portal tracts and distributed near injured interlobular bile ducts (Fig. 2A and B). Notably, the number of CCR6^+^ cells correlated positively with both inflammation severity (r = 0.682, *P* < 0.001) and hepatic fibrosis stage (r = 0.515, *P* = 0.005) in PBC (Fig. 2C and D). These findings suggest that upregulated expression of CCR6 is closely associated with the pathogenesis of PBC.

### 3.5 Association of SNPs in *CCL20* locus with PBC in Han Chinese cohort

Previous GWA studies identified a SNP (rs4973341) in the *CCL*20 locus was significantly associated with PBC (*P* = 2.34 × 10^−10^) in a meta-analysis using European and North American PBC cohorts [11], but none of rs4973341 and SNPs in the shared haplotype with rs4973341 were included in the Human OmniZhongHua-8 Beadchip used in Han Chinese GWAS study. We also noticed that, in the CHB and CHS populations, rs4973341 is in complete linkage disequilibrium with rs10933215, a predicted transcription factor-binding site polymorphism. To further analyze the potential interaction of SNPs in the *CCR6-CCL20* axis, we performed association analysis by genotyping rs10933215 in order to replicate the observed association in European cohorts.

In 1832 PBC cases and 3158 normal controls genotyped for rs10933215, the calculated allelic association is weak, with the *P* value of 0.001 (OR = 0.84, 95% CI = 0.76–0.93), with the C allele as the protective allele (Table 5). This result indicated that the *CCL*20 locus has a weak association with the PBC susceptibility in Han Chinese cohorts. The multiplicative interaction effect of 2-, 3-, 4- and 5-SNPs combinations were tested by binary logistic regression. For 6 interaction combination models, all combinations showed a significant difference between PBC patients and controls (*P* < 0.05, Table S8).

**Table 5:** The genotype and allele frequencies of *CCL20* polymorphisms rs10933215 in PBC patients and controls.

	PBC (MAF)	Control (MAF)	*P*-value	OR (95%CI)
Genotypes				
TT	1244 (0.679)	2023 (0.641)		1.0 (ref.)
TC	534 (0.292)	995 (0.315)	0.035	0.87 (0.77–0.99)
CC	54 (0.029)	140 (0.044)	0.005	0.63 (0.46–0.87)
Alleles				
T	3022 (0.825)	5041 (0.798)		1.0 (ref.)
C	642 (0.175)	1275 (0.202)	0.001	0.84 (0.76–0.93)

MAF, minor allele frequency; OR, Odds ratio; 95% CI, 95% Confidence interval.

## 4 Discussion

In our previous Han Chinese GWAS analysis of 1126 PBC cases and 4036 healthy controls, seventeen SNPs in *CCR6* locus showed variable significance with the calculated *P* value between 1 × 10^−4^ and 2.04 × 10^−7^. These SNPs can be divided as “risk” SNPs and protective SNPs. The most significant SNPs were observed among the “protective” SNPs, which were in close linkage. Subsequent replication of the most significant SNP (rs6456156) in 907 PBC and 2127 controls was not able to replicate the calculated significance. We took a close look at the haplotype structure of those “risk” SNPs and noticed that they can be separated by two LD blocks represented by rs6905911 and rs12529876, respectively. In this report, we were able to replicate their significant association with PBC susceptibility.

In order to further identify the functional variant associated with PBC susceptibility, we noticed a recently identified functional variant (CCR6DNP) in the *CCR6* locus. CCR6DNP is a dinucleotide polymorphism (rs968334 and rs117912866) and associated with genetic susceptibility with rheumatoid arthritis in Japanese cohorts and systemic sclerosis in European cohorts [[22], [23], [24]]. Our results indicated that the TG allele in CCR6DNP is associated with PBC, which corelated with increased *CCR6* expression [22]. The TG allele preferentially binds to poly ADP ribose polymerase 1 (PARP-1) to increase *CCR6* expression, compared to other two alleles.

*CCR6* is mainly expressed in Th17, Treg, and dendritic cells and plays a role in cell recruitment during inflammation. In the inflamed liver of PBC patients, there is increased levels of functional *CCR6* expression on liver infiltrating Th17 cells. *CCL20* is the only ligand for *CCR6* and expressed in inflamed choanocytes of bile ducts [25]. Immunohistochemical examinations found that increased number of *CCR6* expressing intrahepatic CD4^+^ lymphocytes resided in the portal tracts of the inflamed bile ducts in PBC patients, compared to normal livers.

Recently Hitomi and coworkers reported two functional SNPs (rs9459874 and rs1012656) in the *CCR6* locus associated with PBC, based on international *meta*-GWAS results, which included our Han Chinese GWA results [26]. For these two SNPs, rs1012656 is in complete linkage with the “protective” SNPs in our GWA results, which were not replicated in our subsequent validation study. The reported rs9459874 is also in complete LD with our significant “risk” SNP rs77890798, therefore its association with PBC can be represented by functional CCR6DNP.

## 5 Conclusions

In conclusion, our study confirmed the *CCR6* locus is associated with genetic risk with PBC in Han Chinese cohorts. The associated CCR6DNP variant corelated with increased *CCR6* expression, which may lead to increased inflammation in intra-hepatic biliary ducts.

## Funding

This work was supported in part by grants from the 10.13039/501100001809National Natural Science Foundation of China (No. 81870397); 10.13039/501100002949Jiangsu Province Natural Science Fund (No. BK20201131); Shenzhen Kangzhe Pharmaceutical Co. Ltd (URC-126/PBC).

## CRediT authorship contribution statement

**Mingming Zhang:** Writing – original draft, Investigation, Formal analysis. **Zhuye Qin:** Resources. **Yexi Huang:** Investigation. **Wenyan Tian:** Resources. **You Li:** Investigation. **Chan Wang:** Formal analysis. **Weifeng Zhao:** Resources. **Yaping Dai:** Resources. **Xingjuan Shi:** Supervision. **M. Eric Gershwin:** Supervision. **Xiong Ma:** Resources. **Meilin Wang:** Formal analysis. **Xiangdong Liu:** Writing – review & editing, Supervision, Funding acquisition, Conceptualization. **Weichang Chen:** Funding acquisition. **Fang Qiu:** Writing – review & editing, Supervision, Funding acquisition, Conceptualization.

## Declaration of competing interest

The authors declare that they have no known competing financial interests or personal relationships that could have appeared to influence the work reported in this paper.

## Data Availability

Data will be made available on request.

## Abbreviations

AMA: anti-mitochondrial antibody
CCR6: CC chemokine receptor 6
CCL20: CC chemokine ligand 20
CHB: Chinese Han Beijing
CHS: Chinese Han South
CI: confidence interval
DC: dendritic cells
GWA: genome-wide association
GWAS: genome-wide association study
HLA: human leukocyte antigen
LD: linkage disequilibrium
MAF: minor allele frequency
OR: odds ratio
PBC: primary biliary cholangitis
SNP: single-nucleotide polymorphism
Th17: T helper 17
