# Supplementary material for: Association of CCR6 functional polymorphisms with Primary Biliary Cholangitis
Source: J Transl Autoimmun. 2024 Feb 15;8:100234. doi: 10.1016/j.jtauto.2024.100234 (PMC10891324; doi:10.1016/j.jtauto.2024.100234)
Supplement: Multimedia component 1 [file mmc1.docx]

**Supplementary Material**

**Supplementary Tables**

Table S1. Primer and probe sequence in the genotyping of rs12529876, rs4710181 and rs6905911.

| **SNP** | **Primer sequence** | **Probe sequence** | **Length** | **Method** |
| --- | --- | --- | --- | --- |
| rs12529876 | FP: TGGTGCCTAGGAATAACAGACAAG | Probe G: FAM-CTCACACGATGAGCA-MGB | 76bp | TaqMan |
|  | RP: GCAGATATCTAAAAGCAGAGCTAAATAAAG | Probe A: HEX-ACTCACACAATGAGC-MGB |  |  |
|  |  |  |  |  |
| rs4710181 | FP: AATGTCCTTATCTGGTGCAGTCTTT | Probe T: FAM-AACTTCTGGTTCCTGTGA-MGB | 138bp | TaqMan |
|  | RP: CTGACGAATTGCATAGCGCA | Probe C: HEX-AAACTTCTGGTCCCTGTGAT-MGB |  |  |
|  |  |  |  |  |
| rs6905911 | FP: TGGCGACTGCTTCATTTTAAGTT | Probe T: FAM-CAGTTAGAATTTTCTAGCC-MGB | 88bp | TaqMan |
|  | RP: CAGCCATCCAGGAAGAGGG | Probe C: HEX-CAGTTAGAATTCTCTAGCC-MGB |  |  |

Table S2. Allelic association of *CCR6* polymorphisms in the GWAS cohort.

| **Chr** | **Position** | **SNP** | ***P*-value** | **OR (95% CI)** |
| --- | --- | --- | --- | --- |
| Protective |  |  |  |  |
| 6 | 167108812 | rs6456156 | 2.04×10^-7^ | 0.78 (0.71-0.86) |
| 6 | 166946070 | rs2769343 | 2.17×10^-6^ | 0.80 (0.72-0.88) |
| 6 | 167089150 | rs1331301 | 4.10×10^-6^ | 0.80 (0.72-0.88) |
| 6 | 167098278 | rs150110 | 6.07×10^-6^ | 0.80 (0.73-0.88) |
| 6 | 166960220 | rs9366076 | 7.14×10^-6^ | 0.80 (0.73-0.88) |
| 6 | 167041651 | rs162297 | 1.56×10^-5^ | 0.81 (0.74-0.89) |
| 6 | 167113808 | rs6908364 | 2.56×10^-5^ | 0.82 (0.74-0.90) |
| 6 | 167102970 | rs975822 | 4.88×10^-5^ | 0.82 (0.75-0.90) |
| 6 | 167111255 | rs1556413 | 6.60×10^-5^ | 0.82 (0.75-0.91) |
| 6 | 166950460 | rs3798307 | 7.26×10^-5^ | 0.82 (0.75-0.91) |
| 6 | 166947236 | rs1079145 | 8.67×10^-5^ | 0.82 (0.75-0.91) |
| Susceptibility |  |  |  |  |
| 6 | 167108136 | rs6905911 | 1.72×10^-6^ | 1.26 (1.14-1.38) |
| 6 | 167100510 | rs4710181 | 5.62×10^-6^ | 1.24 (1.13-1.37) |
| 6 | 166998128 | rs239936 | 5.96×10^-6^ | 1.24 (1.13-1.36) |
| 6 | 167024500 | rs2301436 | 6.96×10^-5^ | 1.21 (1.10-1.33) |
| 6 | 167048013 | rs12529876 | 7.51×10^-5^ | 1.21 (1.10-1.33) |
| 6 | 167057212 | rs77890798 | 9.52×10^-5^ | 1.21 (1.10-1.33) |

OR, Odds ratio; 95% CI, 95% Confidence interval.

Table S3. The genotypic and allelic association of rs12529876 and rs4710181 and rs6905911 in the replication cohort.

| **SNP** | **Genotypes or alleles** | **PBC cases (MAF)** | **Controls (MAF)** | ***P*-value** | **OR (95%CI)** |
| --- | --- | --- | --- | --- | --- |
| rs12529876 | GG | 648 (0.344) | 1210 (0.387) |  | 1.0 (ref.) |
|  | GA | 905 (0.48) | 1474 (0.471) | 5.59×10^-2^ | 1.12 (0.99-1.26) |
|  | AA | 332 (0.176) | 443 (0.142) | 1.24×10^-5^ | 1.42 (1.22-1.67) |
|  | G | 2201 (0.584) | 3894 (0.623) |  | 1.0 (ref.) |
|  | A | 1569 (0.416) | 2360 (0.377) | 2.76×10^-5^ | 1.18 (1.10-1.27) |
| rs4710181 | TT | 561 (0.302) | 1100 (0.348) |  | 1.0 (ref.) |
|  | TC | 916 (0.493) | 1474 (0.466) | 5.16×10^-4^ | 1.24 (1.10-1.40) |
|  | CC | 381 (0.205) | 590 (0.186) | 3.01×10^-3^ | 1.26 (1.08-1.46) |
|  | T | 2038 (0.548) | 3674 (0.581) |  | 1.0 (ref.) |
|  | C | 1678 (0.452) | 2654 (0.419) | 6.99×10^-4^ | 1.14 (1.06-1.23) |
| rs6905911 | CC | 427 (0.235) | 915 (0.279) |  | 1.0 (ref.) |
|  | CT | 896 (0.493) | 1574 (0.480) | 6.74×10^-4^ | 1.26 (1.10-1.44) |
|  | TT | 494 (0.272) | 790 (0.241) | 8.15×10^-5^ | 1.36 (1.17-1.58) |
|  | C | 1750 (0.482) | 3404 (0.519) |  | 1.0 (ref.) |
|  | T | 1884 (0.518) | 3154 (0.481) | 5.54×10^-5^ | 1.17 (1.08-1.26) |

MAF, minor allele frequency; OR, Odds ratio; 95% CI, 95% Confidence interval. *P* values were calculated using logistic regression model by SPSS 23.0

Table S4. Summary of functional annotation of SNPs which showed r^2^ > 0.8 with rs12529876 from RegulomeDB and HaploReg.

| **chr** | **pos (hg38)** | **LD** | **variant** | **Promoter^a^** | **Enhancer^b^** | **DNAse^c^** | **Proteins** | **Motifs** | **Selected** | **GENCODE** | **dbSNP** | **RegulomeDB rank^f^** |
| --- | --- | --- | --- | --- | --- | --- | --- | --- | --- | --- | --- | --- |
|  |  | **(r²)** |  | **histone marks** | **histone marks** |  | **bound^d^** | **changed^e^** | **eQTL hits** | **genes** | **func annot** |  |
| 6 | 167017659 | 0.93 | rs12526548 |  |  |  |  | AP-1,Sox | 13 hits | FGFR1OP | intronic | 1f |
| 6 | 167018341 | 0.93 | rs9459839 |  |  | BRST |  |  | 12 hits | FGFR1OP | intronic | 1f |
| 6 | 167019278 | 0.93 | rs35171809 |  | ESDR | IPSC |  | 5 altered motifs | 13 hits | FGFR1OP | intronic | 1b |
| 6 | 167021198 | 0.93 | rs1894603 |  |  |  |  |  | 13 hits | FGFR1OP | intronic | 1f |
| 6 | 167024500 | 0.93 | rs2301436 |  |  |  |  | Gfi1 | 14 hits | FGFR1OP | intronic | 7 |
| 6 | 167027013 | 0.93 | rs9459845 |  | IPSC |  |  | 4 altered motifs | 12 hits | FGFR1OP | intronic | 7 |
| 6 | 167027248 | 0.93 | rs9457257 |  | ESC, IPSC | BRN |  |  | 13 hits | FGFR1OP | intronic | 1f |
| 6 | 167027535 | 0.93 | rs9459846 |  | 4 tissues | KID,GI |  | GR,Lmo2-complex | 13 hits | FGFR1OP | intronic | 1f |
| 6 | 167029507 | 0.83 | rs2237274 |  | BLD, VAS |  |  | 4 altered motifs | 13 hits | FGFR1OP | intronic | 1f |
| 6 | 167029529 | 0.93 | rs2237273 |  | BLD |  |  | 5 altered motifs | 13 hits | FGFR1OP | intronic | 1f |
| 6 | 167029914 | 0.93 | rs6456146 |  | BLD |  |  | SIX5 | 14 hits | FGFR1OP | intronic | 7 |
| 6 | 167030874 | 0.82 | rs200447952 |  | 7 tissues | BLD |  | BCL,NRSF | 12 hits | FGFR1OP | intronic | 1f |
| 6 | 167034625 | 0.95 | rs9295384 |  |  | BLD |  | GR,LUN-1,THAP1 | 13 hits | FGFR1OP | intronic | 1f |
| 6 | 167034885 | 0.95 | rs34458103 |  |  |  |  | 8 altered motifs | 1 hit | FGFR1OP | intronic | 7 |
| 6 | 167043372 | 0.88 | rs7748224 |  | BLD |  |  | 7 altered motifs | 10 hits | 953bp 3' of FGFR1OP |  | 1b |
| 6 | 167045090 | 0.95 | rs71032899 |  | MUS |  |  | 14 altered motifs | 1 hit | 2.7kb 3' of FGFR1OP |  | 1f |
| 6 | 167045304 | 0.99 | rs62436765 |  |  | 4 tissues |  |  | 8 hits | 2.9kb 3' of FGFR1OP |  | 1f |
| 6 | 167046108 | 1 | rs10946208 |  |  |  | CTCF,RAD21 | Foxj2,Irx,Pou2f2 | 11 hits | 3.7kb 3' of FGFR1OP |  | 1f |
| 6 | 167046203 | 1 | rs201851004 |  | BLD, GI |  |  | 8 altered motifs | 10 hits | 3.8kb 3' of FGFR1OP |  | 5 |
| 6 | 167047049 | 1 | rs6909180 | BLD | 7 tissues | 5 tissues | CTCF,RAD21 | 4 altered motifs | 11 hits | 4.6kb 3' of FGFR1OP |  | 1f |
| 6 | 167047230 | 1 | rs12213683 | BLD | 7 tissues | BLD,BLD |  | Myc,PTF1-beta,ZNF263 | 12 hits | 4.8kb 3' of FGFR1OP |  | 1b |
| 6 | 167048013 | 1 | **rs12529876** | BLD, GI | BLD, THYM | 4 tissues |  | Sox | 11 hits | 5.6kb 3' of FGFR1OP |  | 1f |
| 6 | 167048157 | 1 | rs12528323 | BLD | BLD, THYM |  |  | NRSF | 11 hits | 5.7kb 3' of FGFR1OP |  | 1f |
| 6 | 167048187 | 0.82 | rs66940113 | BLD | BLD, THYM |  |  | Pax-2 | 10 hits | 5.8kb 3' of FGFR1OP |  | 1f |
| 6 | 167048258 | 1 | rs12528689 | BLD | BLD, THYM |  |  | HES1 | 11 hits | 5.8kb 3' of FGFR1OP |  | 1f |
| 6 | 167048477 | 1 | rs13208636 | BLD | BLD, SKIN |  |  | Zbtb3 | 9 hits | 6.1kb 3' of FGFR1OP |  | 1b |
| 6 | 167050414 | 0.99 | rs6456149 |  | BLD, SKIN | BLD |  | AFP1,Irf | 11 hits | 8kb 3' of FGFR1OP |  | 7 |
| 6 | 167051842 | 1 | rs2181058 |  |  |  |  | Foxp1,Foxp3 | 11 hits | 9.4kb 3' of FGFR1OP |  | 7 |
| 6 | 167052088 | 1 | rs12529238 |  |  |  |  | 5 altered motifs | 11 hits | 9.7kb 3' of FGFR1OP |  | 7 |
| 6 | 167052951 | 1 | rs6932740 |  | BLD |  |  | Osr | 10 hits | 11kb 3' of FGFR1OP |  | 7 |
| 6 | 167052961 | 1 | rs6934043 |  | BLD |  |  |  | 10 hits | 11kb 3' of FGFR1OP |  | 7 |
| 6 | 167053613 | 1 | rs1358882 | BLD | BLD, HRT |  |  | 5 altered motifs | 11 hits | 11kb 3' of FGFR1OP |  | 1f |
| 6 | 167053861 | 1 | rs720325 | BLD | 5 tissues | BLD,SKIN |  | Ik-2 | 10 hits | 11kb 3' of FGFR1OP |  | 1f |
| 6 | 167053945 | 1 | rs1358883 | BLD | 6 tissues | 4 tissues |  |  | 11 hits | 12kb 3' of FGFR1OP |  | 1f |
| 6 | 167054191 | 1 | rs9457261 |  | BLD, HRT, BRST | 4 tissues |  | 4 altered motifs | 11 hits | 12kb 3' of FGFR1OP |  | 1f |
| 6 | 167055171 | 1 | rs1407315 | BLD | BLD | BLD |  | Sin3Ak-20 | 10 hits | 13kb 3' of FGFR1OP |  | 1f |
| 6 | 167055434 | 0.95 | rs6456150 | BLD | BLD |  |  | Roaz | 11 hits | 13kb 3' of FGFR1OP |  | 1f |
| 6 | 167055783 | 0.99 | rs12527827 |  | BLD |  |  | 7 altered motifs | 10 hits | 13kb 3' of FGFR1OP |  | 6 |
| 6 | 167055804 | 1 | rs12529959 |  | BLD |  |  | 4 altered motifs | 10 hits | 13kb 3' of FGFR1OP |  | 7 |
| 6 | 167055993 | 1 | rs6909475 |  | BLD |  |  | 5 altered motifs | 10 hits | 14kb 3' of FGFR1OP |  | 7 |
| 6 | 167056042 | 1 | rs6909502 |  | BLD |  |  | 5 altered motifs | 10 hits | 14kb 3' of FGFR1OP |  | 1f |
| 6 | 167057559 | 1 | rs12523712 |  | ESC, BLD | IPSC,IPSC,THYM |  | 4 altered motifs | 10 hits | 15kb 3' of FGFR1OP |  | 1f |
| 6 | 167057973 | 0.99 | rs911632 |  | BLD |  |  |  | 10 hits | 16kb 3' of FGFR1OP |  | 1f |
| 6 | 167058044 | 0.99 | rs1810644 |  | BLD |  |  | E2A,TBX5,ZEB1 | 10 hits | 16kb 3' of FGFR1OP |  | 1b |
| 6 | 167058145 | 0.92 | rs1810645 |  | BLD |  |  | 5 altered motifs | 9 hits | 16kb 3' of FGFR1OP |  | 1b |
| 6 | 167058226 | 0.99 | rs2017338 |  | BLD |  |  | 5 altered motifs | 10 hits | 16kb 3' of FGFR1OP |  | 1f |
| 6 | 167058363 | 0.99 | rs6456151 |  | BLD, HRT | 8 tissues | CTCF | TCF4,ZNF263 | 11 hits | 16kb 3' of FGFR1OP |  | 1b |
| 6 | 167058679 | 0.99 | rs6456153 |  | BLD, LIV | 4 tissues |  | Hoxd10,STAT | 10 hits | 16kb 3' of FGFR1OP |  | 1f |
| 6 | 167058768 | 0.99 | rs6456154 |  | LIV |  |  | Pou1f1 | 10 hits | 16kb 3' of FGFR1OP |  | 1f |
| 6 | 167059518 | 0.99 | rs12203510 |  | LIV |  | FOXA1 | 5 altered motifs | 11 hits | 17kb 3' of FGFR1OP |  | 1f |
| 6 | 167065387 | 0.96 | rs12525345 |  |  |  |  | 6 altered motifs | 10 hits | 23kb 3' of FGFR1OP |  | 6 |
| 6 | 167066525 | 0.99 | rs12525855 |  |  |  |  | Nr2f2 | 10 hits | 24kb 3' of FGFR1OP |  | 7 |
| 6 | 167067503 | 0.86 | rs10946209 |  |  |  |  | 6 altered motifs | 10 hits | 25kb 3' of FGFR1OP |  | 5 |
| 6 | 167067904 | 0.99 | rs13195158 |  |  |  |  | BCL,Gm397,Nkx2 | 10 hits | 25kb 3' of FGFR1OP |  | 1f |
| 6 | 167068508 | 0.99 | rs2187859 |  |  |  |  | HDAC2 | 11 hits | 26kb 3' of FGFR1OP |  | 7 |
| 6 | 167068521 | 0.97 | rs59889343 |  |  |  |  | 5 altered motifs | 10 hits | 26kb 3' of FGFR1OP |  | 6 |
| 6 | 167069067 | 0.99 | rs9457266 |  |  |  |  | 10 altered motifs | 10 hits | 27kb 3' of FGFR1OP |  | 7 |
| 6 | 167069407 | 0.99 | rs7750209 |  |  |  |  | 6 altered motifs | 10 hits | 27kb 3' of FGFR1OP |  | 7 |
| 6 | 167069747 | 0.99 | rs7746628 |  |  |  |  | 4 altered motifs | 10 hits | 27kb 3' of FGFR1OP |  | 7 |
| 6 | 167070058 | 0.99 | rs12198816 |  |  |  |  | 7 altered motifs | 10 hits | 28kb 3' of FGFR1OP |  | 7 |
| 6 | 167070405 | 0.99 | rs4598049 |  |  |  |  | ZID | 10 hits | 28kb 3' of FGFR1OP |  | 1f |
| 6 | 167070856 | 0.99 | rs2345753 |  |  |  |  | Cdx,Foxp1 | 10 hits | 28kb 3' of FGFR1OP |  | 7 |
| 6 | 167070873 | 0.99 | rs2345754 |  |  |  |  | Foxp1,Pou5f1,Sox | 10 hits | 28kb 3' of FGFR1OP |  | 7 |
| 6 | 167072190 | 0.97 | rs7761977 |  |  |  | SETDB1 | Foxj2,Gm397 | 10 hits | 30kb 3' of FGFR1OP |  | 1f |
| 6 | 167072235 | 0.97 | rs7742305 |  |  |  | SETDB1 | AIRE,BATF,Hoxa5 | 10 hits | 30kb 3' of FGFR1OP |  | 1f |
| 6 | 167072343 | 0.97 | rs7762156 |  |  |  | SETDB1 | Nanog | 10 hits | 30kb 3' of FGFR1OP |  | 1f |
| 6 | 167072730 | 0.97 | rs2001115 |  |  |  |  | Maf,Pou6f1,p300 | 10 hits | 30kb 3' of FGFR1OP |  | 1f |
| 6 | 167073090 | 0.97 | rs2001114 |  |  |  |  | 4 altered motifs | 11 hits | 31kb 3' of FGFR1OP |  | 7 |
| 6 | 167073523 | 0.97 | rs9459862 |  | BLD |  |  | AP-3 | 11 hits | 31kb 3' of FGFR1OP |  | 7 |
| 6 | 167090151 | 0.92 | rs9457268 | BLD | 7 tissues | BLD,BLD |  | TFIIA,ZBTB33 | 17 hits | 22kb 5' of CCR6 |  | 1f |
| 6 | 167090639 | 0.93 | rs9459874 | BLD | 6 tissues | BLD,BLD |  |  | 19 hits | 21kb 5' of CCR6 |  | 1f |
| 6 | 167092060 | 0.92 | rs2285147 | BLD | 8 tissues | 7 tissues |  | RP58 | 18 hits | 20kb 5' of CCR6 |  | 1f |
| 6 | 167092303 | 0.93 | rs6902119 | BLD | 7 tissues | 4 tissues | PAX5C20,POL2 | 5 altered motifs | 19 hits | 20kb 5' of CCR6 |  | 1f |
| 6 | 167092341 | 0.93 | rs6918286 | BLD | 4 tissues | BLD,BLD | PAX5C20,POL2 |  | 19 hits | 19kb 5' of CCR6 |  | 1b |
| 6 | 167093307 | 0.92 | rs7760495 |  |  |  |  |  |  |  |  | 1f |

a, Histone modifications of H3K4me1 and H3K27ac; b, Histone modification of H3K4me3; c, The levels of DNase I hypersensitivity; d, The binding of transcription factor; e, The alteration in regulatory motif; f, Functional prediction scores of each SNP by the RegulomeDB database.

Table S5. Summary of functional annotation of SNPs which showed r^2^ > 0.8 with rs4710181 from RegulomeDB and HaploReg.

| **chr** | **pos (hg38)** | **LD** | **variant** | **Promoter^a^** | **Enhancer^b^** | **DNAse^c^** | **Proteins** | **Motifs** | **Selected** | **GENCODE** | **dbSNP** | **RegulomeDB rank^f^** |
| --- | --- | --- | --- | --- | --- | --- | --- | --- | --- | --- | --- | --- |
|  |  | **(r²)** |  | **histone marks** | **histone marks** |  | **bound^d^** | **changed^e^** | **eQTL hits** | **genes** | **func annot** |  |
| 6 | 167099408 | 0.99 | rs9366089 |  | 5 tissues | BLD |  | Pou1f1 | 5 hits | 12kb 5' of CCR6 |  | 1f |
| 6 | 167099764 | 1 | rs7775443 |  | BLD, PLCNT |  |  | GATA | 5 hits | 12kb 5' of CCR6 |  | 1f |
| 6 | 167100044 | 1 | rs4710178 | BLD | BLD, THYM, PLCNT |  |  | Mxi1,SREBP,Tgif1 | 5 hits | 12kb 5' of CCR6 |  | 1f |
| 6 | 167100204 | 1 | rs4710179 |  | BLD | BLD |  |  | 4 hits | 12kb 5' of CCR6 |  | 1f |
| 6 | 167100510 | 1 | **rs4710181** |  | BLD | BLD | 5 bound proteins |  | 5 hits | 11kb 5' of CCR6 |  | 1f |
| 6 | 167100573 | 1 | rs4710182 |  | BLD | BLD | POL24H8,POL2B | 4 altered motifs | 5 hits | 11kb 5' of CCR6 |  | 1d |
| 6 | 167100595 | 1 | rs4710183 |  | BLD | BLD |  | ATF3 | 5 hits | 11kb 5' of CCR6 |  | 1f |

a, Histone modifications of H3K4me1 and H3K27ac; b, Histone modification of H3K4me3; c, The levels of DNase I hypersensitivity; d, The binding of transcription factor; e, The alteration in regulatory motif; f, Functional prediction scores of each SNP by the RegulomeDB database.

Table S6. Summary of functional annotation of SNPs which showed r^2^ > 0.8 with rs6905911 from RegulomeDB and HaploReg.

| **chr** | **pos (hg38)** | **LD** | **variant** | **Promoter^a^** | **Enhancer^b^** | **DNAse^c^** | **Proteins** | **Motifs** | **Selected** | **GENCODE** | **dbSNP** | **RegulomeDB rank^f^** |
| --- | --- | --- | --- | --- | --- | --- | --- | --- | --- | --- | --- | --- |
|  |  | **(r²)** |  | **histone marks** | **histone marks** |  | **bound^d^** | **changed^e^** | **eQTL hits** | **genes** | **func annot** |  |
| 6 | 167102459 | 1 | rs10946212 |  |  | IPSC | EBF1 | GATA,HNF4 | 5 hits | 9.3kb 5' of CCR6 |  | 1f |
| 6 | 167102607 | 0.99 | rs10946213 |  |  | BLD |  | GR | 5 hits | 9.2kb 5' of CCR6 |  | 1f |
| 6 | 167102634 | 1 | rs10946214 |  |  |  |  | 4 altered motifs | 5 hits | 9.2kb 5' of CCR6 |  | 1f |
| 6 | 167102693 | 1 | rs10946215 |  |  |  |  | PRDM1 | 5 hits | 9.1kb 5' of CCR6 |  | 1f |
| 6 | 167102811 | 1 | rs2039321 |  | 4 tissues | BLD |  | 4 altered motifs | 5 hits | 9kb 5' of CCR6 |  | 1f |
| 6 | 167104984 | 0.98 | rs7765739 |  | BLD | LNG |  | 4 altered motifs | 4 hits | 6.8kb 5' of CCR6 |  | 1f |
| 6 | 167106298 | 1 | rs7774396 |  | BLD | BLD,LNG |  | 4 altered motifs | 4 hits | 5.5kb 5' of CCR6 |  | 1f |
| 6 | 167106419 | 0.85 | rs7761159 |  | BLD | SKIN |  | 9 altered motifs | 6 hits | 5.4kb 5' of CCR6 |  | 1f |
| 6 | 167107261 | 1 | rs9457273 |  | BLD |  |  | 5 altered motifs | 5 hits | 4.5kb 5' of CCR6 |  | 6 |
| 6 | 167107377 | 0.9 | rs9457274 |  | BLD |  |  | Hmbox1 | 7 hits | 4.4kb 5' of CCR6 |  | 1f |
| 6 | 167108074 | 1 | rs6905876 |  | BLD | BLD | BATF |  | 3 hits | 3.7kb 5' of CCR6 |  | 1f |
| 6 | 167108136 | 1 | **rs6905911** |  | BLD |  |  | Cdx2,Hsf | 3 hits | 3.7kb 5' of CCR6 |  | 1f |
| 6 | 167108188 | 0.9 | rs4709148 |  | BLD |  |  | 6 altered motifs | 4 hits | 3.6kb 5' of CCR6 |  | 1f |
| 6 | 167108812 | 0.9 | rs6456156 |  | BLD, PLCNT |  |  | GR | 6 hits | 3kb 5' of CCR6 |  | 1f |
| 6 | 167110751 | 0.9 | rs2021033 | BLD | ESDR, BLD |  |  |  | 3 hits | 1.1kb 5' of CCR6 |  | 1f |

a, Histone modifications of H3K4me1 and H3K27ac; b, Histone modification of H3K4me3; c, The levels of DNase I hypersensitivity; d, The binding of transcription factor; e, The alteration in regulatory motif; f, Functional prediction scores of each SNP by the RegulomeDB database.

Table S7. Summary of functional annotation of SNPs which showed r^2^ > 0.8 with rs968334 from RegulomeDB and HaploReg.

| **chr** | **pos (hg38)** | **LD** | **variant** | **Promoter^a^** | **Enhancer^b^** | **DNAse^c^** | **Proteins** | **Motifs** | **Selected** | **GENCODE** | **dbSNP** | **RegulomeDB rank^f^** |
| --- | --- | --- | --- | --- | --- | --- | --- | --- | --- | --- | --- | --- |
|  |  | **(r²)** |  | **histone marks** | **histone marks** |  | **bound^d^** | **changed^e^** | **eQTL hits** | **genes** | **func annot** |  |
| 6 | 167112608 | 1 | **rs968334** | BLD, GI |  | BLD,BLD |  | NRSF,Zfp740 | 7 hits | CCR6 | intronic | 1b |
| 6 | 167119243 | 0.8 | rs3093025 | BLD | 5 tissues | BLD,BLD,BLD |  | HNF4 | 6 hits | CCR6 | intronic | 1f |
| 6 | 167119305 | 0.8 | rs3093024 | BLD | 5 tissues | ESC,BLD,BLD |  |  | 6 hits | CCR6 | intronic | 1f |
| 6 | 167120802 | 0.8 | rs3093023 | BLD, GI | 5 tissues | 8 tissues |  | 5 altered motifs | 6 hits | CCR6 | intronic | 1f |

a, Histone modifications of H3K4me1 and H3K27ac; b, Histone modification of H3K4me3; c, The levels of DNase I hypersensitivity; d, The binding of transcription factor; e, The alteration in regulatory motif; f, Functional prediction scores of each SNP by the RegulomeDB database.

Table S8. Multiplicative interaction analysis of gene–gene interactions involved in PBC by logistic regression.

| **Interaction** | **Chi-square** | ***P*-value** | **OR (95% CI)** |
| --- | --- | --- | --- |
| rs10933215×rs12529876 | 15.045 | 1.05 × 10^-4^ | 1.10 (1.05-1.15) |
| rs10933215×rs6905911 | 21.783 | 3.05 × 10^-6^ | 1.11 (1.06 -1.16) |
| rs10933215×rs968334 | 12.335 | 4.44 × 10^-4^ | 1.13 (1.06-1.21) |
| rs10933215×rs4710181 | 11.582 | 0.001 | 1.08 (1.03-1.13) |
| rs10933215×rs6905911×rs968334 | 9.324 | 0.002 | 1.06 (1.02-1.10) |
| rs10933215×rs12529876×rs6905911×rs968334 | 6.599 | 0.01 | 1.03 (1.01-1.05) |
| rs10933215×rs12529876×rs4710181×rs6905911×rs968334 | 5.133 | 0.023 | 1.02 (1.00-1.03) |

OR, Odds ratio; 95% CI, 95% Confidence interval. CCL20: rs10933215; CCR6: rs12529876, rs6905911, rs968334, rs4710181.

**Supplementary Figure**

Figure S1. Locus specific plots for SNPs in *CCR6* locus based on the GWAS data.


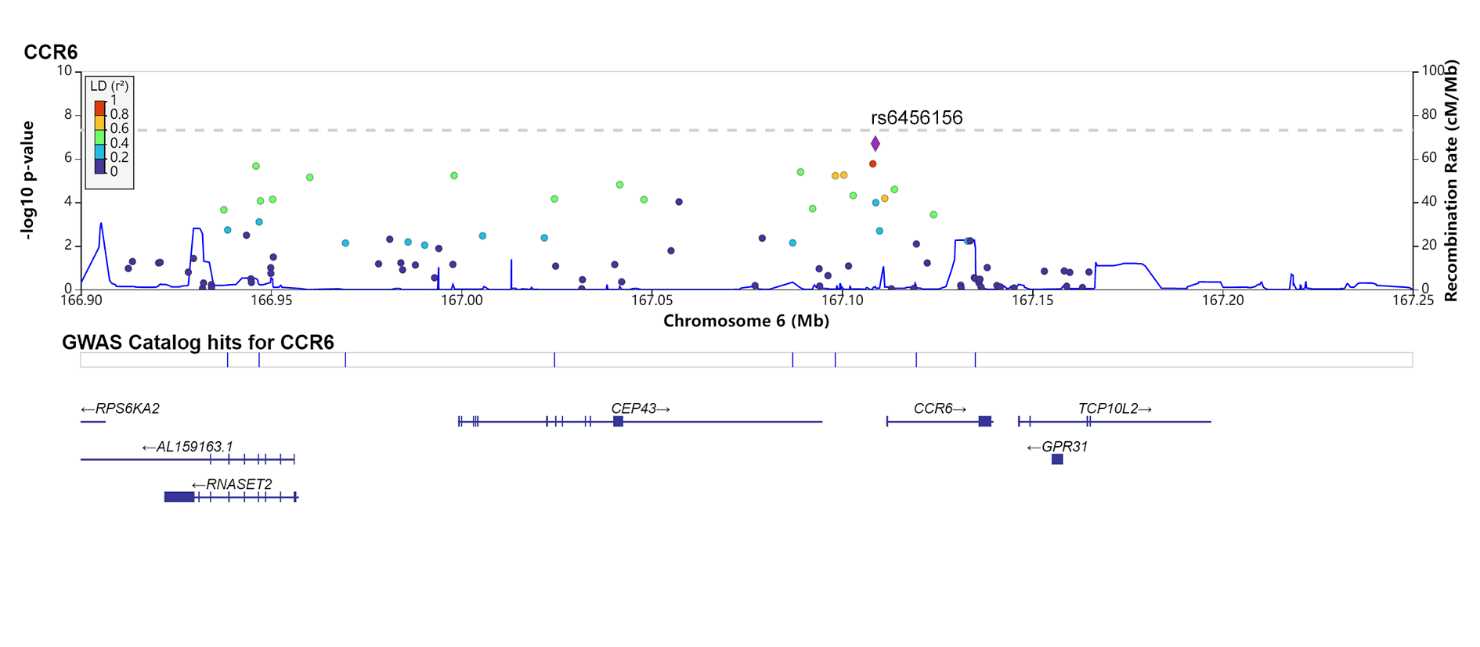


The LocusZoom plots of GWAS *P* value and LD (r^2^) of SNPs with the most significant SNP are shown by the color codes (see legend) depending on their expected degree of correlation (r^2^) with the top SNP.
